# Attitudes towards being offered a choice of self-sampling or clinician sampling for cervical screening: A cross-sectional survey of women taking part in a clinical validation of HPV self-collection devices

**DOI:** 10.1177/09691413241283356

**Published:** 2024-10-09

**Authors:** Laura Marlow, Hannah Drysdale, Jo Waller

**Affiliations:** 1 Centre for Cancer Screening, Prevention and Early Diagnosis, Wolfson Institute of Population Health, 4617 Queen Mary University of London, London, UK; 2 School of Cancer and Pharmaceutical Sciences, 4616 King's College London, London, UK

**Keywords:** HPV, human papillomavirus, decision-making, public attitudes, cancer screening

## Abstract

**Objectives:**

Primary human papillomavirus (HPV) testing in cervical screening offers the opportunity for women to be given a choice between HPV self-sampling and traditional clinician screening. This study assessed attitudes towards a choice and anticipated future preference among women who had collected a vaginal self-sample alongside their usual cervical screen.

**Setting:**

Thirty-eight general practices across five areas in England.

**Methods:**

Overall, 2323 women (24–65 years; response rate: 48%) completed a survey after collecting a self-sample and having a clinician screen at their GP practice. We asked which test they preferred and assessed attitudes to being offered a choice. We explored age, education, ethnicity and screening experience as predictors of attitudes towards a choice and anticipated future choice.

**Results:**

Most participants felt they would like a choice between self-sampling and clinician screening (85%) and thought this would improve screening for them (72%). However, 23% felt it would be difficult to choose, 15% would worry about making a choice, and nearly half would prefer a recommendation (48%). Compared with women with degree-level education, those with fewer qualifications were more likely to say they would worry about having a choice or would not want a choice (p < 0.001). The majority said they would choose to self-sample at home if offered a choice in the future (69%; n = 1602/2320).

**Conclusions:**

Self-sampling is likely to be popular, but offering a choice could cause worry for some people and many would prefer a recommendation. Supporting people to make a choice will be important, particularly for those with lower levels of education.

## Background

The NHS Cervical Screening Programme offers human papillomavirus (HPV)-based screening to all women and people with a cervix between 25 and 64 years, with screening intervals of 3 or 5 years depending on age.^1^ The test procedure involves a speculum examination, usually carried out by a nurse or general practitioner (GP) in a primary care setting. In England, cervical screening coverage is at an all-time low, with more than 3 out of 10 women overdue for their test.^2^ It is well-established that emotional factors (e.g. embarrassment about having the test) and practical considerations (e.g. inconvenient appointment times) influence screening attendance.^3–7^

HPV vaginal self-sampling offers promise as a way of overcoming some of these barriers. There is good evidence that it can increase participation in non-attenders,^8–13^ but the impact on uptake varies depending on how the self-sampling offer is made (e.g. opt-in strategies vs. mailed-out kits).^8,14^ An increasing number of countries, including Australia, the Netherlands and Sweden, are now offering all screening invitees the option of self-sampling,^15–17^ while England is taking a more cautious approach and has been carrying out a clinical validation study ahead of any implementation.^18^ It is too early to fully understand the impact of offering self-sampling in the countries now doing so, but there is an indication that the impact on overall screening coverage has not been as great as expected. In addition, there are concerns that the overall impact of self-sampling on screening programmes may not be uniformly positive, and will depend on test performance relative to clinician sampling (e.g. potentially lower sensitivity), as well as the behaviour of the invited population (e.g. rates of switching from clinician to self-sampling among regular screeners and rates of attendance for clinical follow up in those testing positive for HPV on a self-sample).^19^

It is also possible that the choice itself will have an impact on decision-making and behaviour. One study in the US found that offering under-screened women a choice of self-collection or clinician sampling helped to increase screening participation five-fold, but this was a small-scale study involving door-to-door community engagement.^20^ In the bowel screening context, a study from Poland observed that offering a combination, or choice, of screening test encouraged more under-screened individuals to participate^21^ but the impact of introducing choice to people who already participate in screening regularly is unknown.

To date, no screening programme in England has offered a choice between different test procedures. There is a need to further explore how people feel about being offered a choice of different tests as part of their cervical screening invitation to inform communication strategies. To better plan any future implementation of self-sampling choice, we also need to understand what proportion of regular screening attenders are likely to choose self-sampling if it were an option. Previous research suggests this may be as high as 40%.^22^ We also need to understand any demographic patterning of attitudes and preferences to mitigate any potential widening of disparities in screening uptake.

As part of a national clinical validation of three HPV self-sampling devices, we collected data on attitudes to the future offer of a choice among women who had completed a self-sample alongside their routine screening. We also collected information about which mode of test women preferred and would choose in the future. We aimed to answer the following research questions:

1. How acceptable is the offer of a choice of self-sampling vs. clinician sampling to attenders of cervical screening (including cognitive and emotional aspects of acceptability)?
2. Are there demographic sub-groups for whom the offer of a choice is less acceptable?
3. What proportion of women who have taken part in both clinician screening and self-sampling would choose to do a home-based self-sample in the future if offered a choice?
4. Are there demographic sub-groups who are more likely to say they would choose self-sampling, if offered a choice in the future?

## Methods

### Participants

The study was nested within HPValidate,^18^ a clinical validation of three HPV self-sampling devices carried out by the Department of Health and Social Care in England in 2021–2023. Participants were recruited when attending for routine cervical screening in primary care in five areas of England: London, Bristol, Manchester, Gateshead, and Norfolk & Norwich. Participants were asked to complete a self-sample, using one of three devices, before having their routine screening. After both tests had taken place, the participant was asked to complete an online questionnaire.

### Procedure

Questionnaires were completed electronically on the SurveyMonkey platform accessed using a link on a device that was handed to women in the surgery. The option to send a survey link to an individual's personal device was made available part way through the data collection period following issues with Wi-Fi at some sites. This approach avoided the need for management of paper surveys and data entry. It also meant responses were confidential and not accessible to the sample-taker or anyone else in the GP surgery. Questionnaire data were collected anonymously and were accessed only by the research team.

Surveys were completed between June 2021 and July 2023. Consent for completion of the survey was included in the HPValidate study consent form. The study was approved by the London-Stanmore Research Ethics Committee (ref: 20/LO/1009).

### Measures

The full survey is available at https://osf.io/txawj and was developed in collaboration with the HPValidate steering group, which included a representative of Jo's Cervical Cancer Trust as well as clinicians and other screening experts. The survey included user experience items which are not reported here. After the user experience questions, participants were asked which test they would choose ‘*if offered a choice between doing a self-test at home or having your cervical screening done by a nurse or doctor*’ with the response options: ‘*I would do the self-test at home*’; ‘*I would go for cervical screening with a nurse or doctor*’; ‘*not sure*’; ‘*I wouldn’t have screening*’.

At the end of the survey, participants were given the opportunity to answer eight additional questions assessing how they would feel about being offered a choice between self-sampling and clinician screening. These questions were based on components from the Theoretical Framework of Acceptability (TFA)^23^ that were considered relevant to acceptability of being offered a choice. This included *affective attitude* (‘I would like to be offered a choice…’ and ‘I would feel worried about being offered a choice…’); *intervention coherence* (‘Being offered a choice […] makes sense to me’); *self-efficacy* (‘I would find it difficult to choose…’); *ethicality* (‘… I would assume it was a way of saving the NHS money’); and *perceived effectiveness* (‘A choice […] would improve cervical screening for me’). These items drew on a theoretically informed questionnaire assessing acceptability of health interventions^24^ and previous work assessing acceptability of extended screening intervals.^25^ We also included two items designed to assess preferences for a recommendation over a choice: ‘I would not want to be offered a choice…’ and ‘I would prefer to have a recommendation’.^26^ (Full item wording is included in the footnotes to Table 2).

Socio-demographic questions assessed age, marital status, and sexual orientation. Highest educational qualification was recoded into three levels: low-level (no qualifications or GCSEs/O-levels or equivalent); Mid-level (AS, A-Levels or equivalent, NVQ or equivalent including BTEC General/National, OND or ONC, City and Guilds Craft); High-level (Degree or above, including HND or HNC, NQV level 4 and above, teaching or nursing degree). Ethnicity was assessed using the same wording and categories as the UK Census, 2021^27^ and was recoded into broad categories (White, Black, Asian, mixed and other). Number of previous cervical screens (none, 1, 2 or 3+) and experience of colposcopy (yes, no, unsure and prefer not to say) were also assessed.

No information was provided about the accuracy of HPV self-sampling within the survey, but the participant information sheet for HPValidate stated that the clinical validation study was needed in order to ‘be sure that a self-test is as accurate as a sample taken by a doctor or nurse’ ahead of any implementation within the screening programme.

### Analyses

Analyses were carried out in SPSS v28.

We report the percentage of participants who agreed/strongly agreed with each of the eight attitude items (with 95% confidence intervals (CI)). We examined differences by age, education, ethnicity, sexual orientation and screening experience in the proportion of women who agreed/strongly agreed with each of the eight attitude items (vs. those who disagreed/strongly disagreed or had no opinion). Chi-square tests were used and a p-value of < 0.001 was considered to indicate statistical significance (following a Bonferroni adjustment to allow for 40 comparisons, i.e., 5 independent and 8 dependent variables).

We report the percentage of participants who anticipate that their future choice would be: (1) self-sampling at home; (2) clinician screening and(3) not sure/would not have screening (with 95% CIs). We used logistic regression to explore whether socio-demographic characteristics were associated with anticipated choice of self-sampling (vs. clinician screening), excluding those who were not able to make a choice.

Sensitivity analyses were carried out adjusting for the self-collection device that women used. This variable did not affect the findings, so we have reported unadjusted analyses.

## Results

### Participant characteristics

Of the 4839 women who were eligible for HPValidate, consented and completed a self-sample in the primary care arm, 2323 (48.0%) also completed a survey. Three cases were excluded because their self-reported age was outside the screening eligible age (i.e. < 24 or >65 years) and a further 105 participants (4.5%) chose not to complete any items assessing attitudes to a choice. For the analysis of attitudes to a choice, data for n = 2179–2215 were included, depending on the item. Data for 2320 participants have been included in the analyses of anticipated choice. Sample characteristics are shown in Table 1.

**Table 1.** Characteristics of the sample.

	Preference outcomes (n = 2320)	Attitude outcomes* (n = 2215)
Age in years (mean; SD)	41.14; 10.6	41.08; 10.58
**Age group** (n; %)		
24–29 years	348 (15.0)	341 (15.4)
30–39 years	705 (30.4)	696 (31.4)
40–49 years	621 (26.8)	605 (27.3)
50–59	414 (17.8)	405 (18.3)
60+	130 (5.6)	123 (5.6)
*Missing data*	102 (4.4)	45 (2.0)
**Marital status** (n; %)		
Single	527 (22.7)	519 (23.4)
Married/civil partnership/cohabiting	1522 (65.6)	148 (67.2)
Separated/divorced/widowed	180 (7.8)	172 (7.8)
*Missing data*	91 (3.9)	36 (1.6)
**Educational level ^a^** (n; %)		
Low-level	273 (11.8)	260 (11.7)
Mid-level	635 (27.4)	621 (28.0)
High-level	1267 (54.6)	1247 (56.3)
*Missing data*	145 (6.3)	87 (3.9)
**Sexual orientation** (n; %)		
Heterosexual/straight	2085 (89.9)	2036 (91.9)
Gay, lesbian, bisexual, other	125 (5.4)	123 (5.6)
*Missing data*	110 (4.7)	56 (2.5)
**Ethnic background** (n; %)		
White	1991 (85.8)	1951 (88.1)
Mixed	57 (2.5)	55 (2.5)
Asian	104 (4.5)	102 (4.6)
Black	59 (2.5)	54 (2.4)
Other	33 (1.4)	31 (1.4)
*Missing data*	76 (3.3)	22 (1.0)
**Screening status** (n; %)		
Today is my first screen	200 (8.6)	193 (8.7)
One previous screen	245 (10.6)	240 (10.8)
Two previous screens	191 (8.2)	189 (8.5)
Three or more previous screens	1561 (67.3)	1526 (68.9)
*Missing data*	123 (5.3)	67 (3.0)
**Experience of colposcopy** (n; %)		
Yes	513 (22.1)	502 (22.7)
No	1750 (75.4)	1710 (77.2)
*Missing data*	57 (2.5)	3 (0.1)

Note: ‘Missing data’ include those who selected ‘prefer not to say’ or were unsure. All demographic/experience data were missing for n = 54. These participants have not been included in the demographic analyses.

* Completed at least one of the attitude items.

a See Methods section for education level details.

### Attitudes to being offered a choice between clinician or self-sampling

Most participants felt that they would like to be offered a choice (84.9%, 95% CI 83.3–86.3%), felt a choice made sense to them (86.3%, 95% CI 84.8–87.7%) and felt that being offered a choice would improve cervical screening for them (71.7%, 95% CI 69.8–73.5%) (Table 2). Overall, 12.4% (95% CI 11.1–13.8%) said they would not want a choice and 15.1% (95% CI 13.6–16.6%) would be worried about a choice. Half felt they would want a recommendation to do either self-sampling or have a clinician test (48.3%, 95% CI 46.2–50.4%), and 22.8% (95% CI 22.1–24.6%) would find it difficult to choose. A significant proportion believed that the offer of a choice would be a way of saving the NHS money (41.6%, 95% CI 39.5–43.6%) but the question wording did not allow us to interpret whether this was viewed as positive or negative.

**Table 2.** Percentage agreement (agree/strongly agree) and socio-demographic correlates of agreement with each attitude item.

	Item 1 makes sense		Item 2 like a choice		Item 3 improve screening		Item 4 prefer recommendation		Item 5 money-saving		Item 6 difficult to choose		Item 7 worried		Item 8 not want choice	
	%	χ^2^ p	%	χ^2^ p	%	χ^2^ p	%	χ^2^ p	%	χ^2^ p	%	χ^2^ p	%	χ^2^ p	%	χ^2^ p
**Overall agreement ^a^**	**86**.**3**		**84**.**9**		**71**.**7**		**48**.**3**		**41**.**6**		**22**.**8**		**15**.**1**		**12**.**4**	
**Age group**																
24–29 years	87.1	0.825	87.1	0.802	73.8	0.878	53.7	0.166	34.3	0.002	25.1	0.439	13.5	0.171	10.9	0.032
30–39 years	85.6		84.3		71.1		46.0		41.3		21.6		14.4		9.5	
40–49 years	85.9		85.0		71.6		47.4		41.0		21.2		13.5		13.1	
50–59	87.8		84.3		72.3		47.5		44.5		24.3		16.5		14.6	
60+	87.7		85.8		69.7		52.5		54.1		26.2		21.3		17.1	
**Educational level ^b^**																
Low-level	84.2	0.264	82.2	0.041	72.1	0.034	48.2	0.748	47.7	0.016	24.5	0.283	21.0	**<0**.**001**	19.8	**<0**.**001**
Mid-level	86.5		83.8		68.6		49.9		43.8		24.2		16.6		16.4	
High-level	87.8		87.1		74.3		48.1		39.1		21.4		12.4		8.6	
**Sexual orientation**																
Heterosexual/straight	86.6	0.127	84.9	0.084	71.4	0.002	48.5	0.316	42.0	0.305	23.0	0.114	15.4	0.076	12.4	0.220
Gay, lesbian, bisexual, other	91.8		91.1		84.6		43.4		36.9		16.4		9.0		8.2	
**Ethnic background**																
Any White background	86.7	0.638	85.2	0.631	71.9	0.852	47.6	0.040	40.2	**<0**.**001**	22.6	0.441	14.3	0.013	11.6	0.016
Any other background	85.4		83.8		72.7		54.8		53.6		25.0		20.6		17.3	
**Screening status**																
Today is my first screen	91.1	0.103	90.7	0.015	75.5	0.029	57.5	0.051	30.9	0.002	28.6	0.180	11.5	0.580	11.4	0.740
One previous screen	83.3		87.0		78.2		46.6		40.3		23.5		14.3		10.9	
Two previous screens	84.7		79.4		74.1		46.8		35.1		21.2		15.3		14.3	
Three or more previous screens	86.5		84.7		70.0		47.1		43.5		21.8		15.2		12.0	

Note: Bold indicates significance at p < 0.001.

a 95% confidence intervals are given in the text.

b See Methods section for education level details.

Item 1: Being offered a choice between self-testing and clinician testing for cervical screening makes sense to me (n = 2207).

Item 2: I would like to be offered a choice between self-testing and clinician testing for cervical screening (n = 2208).

Item 3: Offering a choice between self-testing and clinician testing would improve cervical screening for me (n = 2205).

Item 4: I would prefer to have a recommendation to do either self-testing or clinician testing rather than having to make a choice myself (n = 2206).

Item 5: If I was given the choice between self-testing and clinician testing for cervical screening, I would assume it was a way of saving the NHS money (n = 2204).

Item 6: I would find it difficult to choose between self-testing and clinician testing for cervical screening (n = 2203).

Item 7: I would feel worried about being offered a choice between self-testing and clinician testing for my cervical screening (n = 2201).

Item 8: I would not want to be offered a choice between self-testing and clinician testing for my cervical screening (n = 2199).

We looked at whether agreeing with any of the attitude items was statistically significantly (p > 0.001) associated with age, education level, ethnicity, sexual orientation or screening status (see Table 3). Participants with a lower level of education were more likely to say they would worry about being offered a choice (21.0% vs. 12.4% of those with high-level education) and more likely to say they would not want to be offered a choice (19.8% vs. 8.6% of those with high-level education). Participants from ethnic minority backgrounds were more likely to think the offer of self-sampling was a way for the NHS to save money (53.6% vs. 40.2% of those from White backgrounds).

**Table 3.** Logistic regression analysis of socio-demographic correlates of anticipated choice to complete a self-sampling test at home if this was offered in the future (n = 1992).

	n (%) who would choose to do self-sample at home	Odds of selecting self-sample (vs clinician test)	
		χ^2^ (df), p-value	Odds ratio (95% CI)
**Age group**			
24–29 years	233 (76.1)	χ^2^(4) = 9.85, 0.043	1.00 (0.73–1.39)
30–39 years	461 (76.1)		Ref
40–49 years	454 (82.2)		**1.46** (**1.09–1.94)**
50–59	299 (81.7)		**1.40** (**1.02–1.94)**
60+	96 (80.0)		1.26 (0.78–2.04)
**Marital status**			
Single	360 (77.9)	χ^2^(2) = 0.94, 0.627	0.94 (0.73–1.21)
Married/civil partnership/cohabiting	1059 (79.0)		Ref
Separated/divorced/widowed	132 (81.5)		1.17 (0.77–1.78)
**Educational level ^a^**			
Low-level	183 (75.3)	χ^2^(3) = 12.46, 0.002	**0.67** (**0.48–0.93)**
Mid-level	416 (75.4)		**0.67** (**0.52–0.86)**
High-level	921 (82.0)		Ref
**Sexual orientation**			
Heterosexual/straight	1444 (78.9)	χ^2^(1) = 0.61, 0.435	Ref
Gay, lesbian, bisexual, other	95 (81.9)		1.21 (0.74–1.97)
**Ethnic background**			
White	1383 (79.2)	χ^2^(4) = 6.57, 0.161	Ref
Mixed	46 (88.5)		2.01 (0.85–4.75)
Asian	67 (72.0)		0.68 (0.42–1.08)
Black	40 (74.1)		0.75 (0.40–1.39)
Other	21 (77.8)		0.92 (0.37–2.29)
**Screening status**			
Today is my first screen	140 (79.5)	χ^2^(3) = 3.22, 0.359	0.99 (0.68–1.47)
One previous screen	159 (75.0)		0.77 (0.55–1.08)
Two previous screens	129 (75.9)		0.81 (0.55–1.17)
Three or more previous screens	1098 (79.6)		Ref
**Experience of colposcopy**			
Yes	321 (72.5)	χ^2^(1) = 12.81, < 0.001	**0.64 (0.50–0.81)**
No	1245 (80.5)		Ref

Univariate analyses reported. Denominator excludes those who did not provide answers for any of the socio-demographic or experience variables (n = 54) and participants who were unsure about their future screening choice or would not have screening (n = 274). Significant differences (p < 0.05) are shown in bold.

^a^ See Methods section for education level details.

### Anticipated future choice

When asked which they would choose in the future if they were offered a choice between doing a self-sample at home or having cervical screening done by a nurse or doctor, the majority said they would choose a self-sample (69.1%; 95% CI 67.1–70.9%; n = 1602/2320) and 18.7% (95% CI 17.2–20.3%) would choose screening done by a nurse or doctor (434/2320). The remaining 12.2% (95% CI 11.0–13.6%) were not sure (n = 281) or would not have screening (n = 3).

We explored socio-demographic correlates of anticipated choice to do a self-sample at home if a choice were offered in the future (see Table 3). Age (χ^2^(4) = 9.85, p = 0.043) and education level (χ^2^(2) = 12.46, p = 0.002) were significantly associated with choosing a self-sample compared to clinician screening. Participants in older age groups (40–49 and 50–59 years) were more likely to say they would choose a self-sample (82.2% and 81.7%, respectively) than participants aged 30–39 years (76.1%). Participants with low- and mid-level education were less likely to say they would choose a self-sample (75.3% and 75.4%, respectively) than those with high-level education (82.0%). Participants with previous experience of colposcopy were less likely to say they would choose a self-sample than those without (72.5% vs. 80.5%; χ^2^(1) = 12.81, p < 0.001).

## Discussion

Many previous studies have examined preferences for HPV self-sampling versus clinician-sampling in the context of cervical screening but, to our knowledge, this is the first study to explore attitudes towards the choice itself. Attitudes towards having a choice were broadly positive but almost half the sample would prefer to have a recommendation than make a choice, and a significant minority said they would find it hard to choose or would be worried about making a choice.

To date, there has been an implicit assumption in screening policy-making that offering self-sampling as a choice would represent an improvement to the service and would be welcomed by the eligible population. Our findings confirm that a choice would be welcomed by most, but the preference for a recommendation among half the sample is somewhat contradictory. Previous research has found that when it comes to taking part in screening, most people would prefer to have a recommendation to participate rather than being asked to evaluate the information and make a decision themselves^28,29^ and only 15% of adults said they would prefer to ‘analyse and choose’ whether to take part in colorectal screening without a recommendation from the NHS.^29^ A choice between two types of screening test arguably constitutes a more complex and nuanced decision than the choice to be screened or not, so it is perhaps not surprising that a recommendation would be valued here too. Early experience from the cervical screening programme in the Netherlands suggests that offering a choice may not increase screening participation, in contrast to expectations.^30^ The difficulty of making a choice may be a contributing factor.

Our findings point to the need for careful consideration of the way in which any offer of a choice of self-sampling is made, to avoid a decrease in participation; and a potential widening of health inequalities if a choice is more off-putting to less educated groups who are already at greater risk of non-participation. Decision-support tools could be one way of helping people to make an informed choice, but they rely on people being willing to engage with the information and to weigh up the pros and cons.^31^ It may also be appropriate to present either self-sampling or clinician screening as the default option, to overcome decision inertia in people who are positively inclined to participate but are unsure which option to choose. Using this kind of behavioural ‘nudge’ in the context of a screening programme could be seen as problematic in that it potentially undermines people's decision-making autonomy,^32^ but it may be acceptable if accompanied by clear signposting to additional information for those who would like it.

Over a fifth of participants said they would find the choice difficult, but our study does not shed light on the reasons for this. Further work using qualitative methods or more detailed survey items would be useful to better understand this and could feed into the development of appropriate information and support materials. Women in our study were not provided with any information about the relative accuracy of self- versus clinician-sampling, so they may have felt insufficiently informed to make a choice, which could only be based on their experience of doing the two tests.

Reassuringly, there were relatively few demographic differences in attitudes towards a choice. Women from ethnic minority backgrounds were more likely to believe a choice is designed to save the NHS money, but it is unclear whether this would be viewed positively or negatively. Of greater concern, women with lower levels of education were more likely to say a choice would be worrying or that they would not want a choice. Reasons for this need to be understood in order to ensure that any implementation of a choice does not disproportionately worry or disengage those who are already less likely to participate.

Around 70% of participants (all of whom had attended for routine screening in primary care and also collected a self-sample) said they would choose self-sampling if they were offered it in the future. This was somewhat higher than has been found in some previous studies,^22,33^ including a global review which identified a pooled estimate of 59% of women preferring self-sampling (over clinician screening). Our study also identified higher self-sampling preferences in older women, consistent with previous research suggesting that self-sampling preferences for older women may be driven by the fact that speculum examinations tend to be more painful after the menopause.^3,34^ Self-sampling was less frequently preferred by women with fewer educational qualifications, echoing our previous study.^22^ It is well-established that women from low socioeconomic status backgrounds have lower levels of health literacy^35^ and this may have contributed to a preference for the screening status quo.

### Strengths and limitations

Our study benefitted from a large and reasonably diverse sample of screening attenders in England, including women with and without previous experience of screening; however, women from ethnic minority groups were somewhat under-represented. We did not include any screening non-attenders which limits the extent to which our findings can be generalised to the wider screening-eligible population whose attitudes to a choice may differ. In addition, since all our participants had agreed to try a self-sample for HPValidate it is likely that those included were already open to the idea of self-sampling. The findings are also unlikely to reflect how people without any experience of trying a self-sample will feel about the choice. Uncertainty around the accuracy of HPV self-sampling was implicit in the participant information materials for HPValidate and this may have affected women's attitudes. Providing evidence-based information about the relative accuracy of the two sampling methods will be essential as part of any future evaluation or implementation.

Our measures were unvalidated but were designed to address a range of attitudes towards a choice, informed by the TFA.^23^ We had input from a stakeholder group including representatives from the NHS Cervical Screening Programme and a cervical cancer charity but did not pilot test the survey or carry out formal patient and public involvement work as part of its development.

## Conclusion

The majority of women who currently attend cervical screening are likely to welcome a choice of clinician or self-sampling for HPV testing but almost half would rather have a recommendation than make a choice themselves. This suggests an urgent need to develop and test different ways of offering this choice so that optimal messaging can be used in any roll-out or evaluation of HPV self-sampling choice in the United Kingdom and elsewhere.
